# mTOR inhibitors potentially reduce TGF-β2-induced fibrogenic changes in trabecular meshwork cells

**DOI:** 10.1038/s41598-021-93580-3

**Published:** 2021-07-08

**Authors:** Nozomi Igarashi, Megumi Honjo, Makoto Aihara

**Affiliations:** Department of Ophthalmology, Graduate School of Medicine, The University of Tokyo, 7-3-1 Hongo Bunkyo-ku, Tokyo, 113-8655 Japan

**Keywords:** Eye diseases, Optic nerve diseases

## Abstract

We examined the effects of mTOR inhibitors on the fibrotic response induced by transforming growth factor-beta2 (TGF-β2) in cultured human trabecular meshwork (hTM) cells. TGF-β2-induced expression of fibronectin, collagen type I, alpha 1 chain (COL1A1), and alpha-smooth muscle actin (αSMA) in hTM cells was examined in the presence or absence of mTOR inhibitors using quantitative real-time polymerase chain reaction, Western blotting, and immunohistochemistry. The migration rates of hTM cells were examined in the presence of TGF-β2 with or without mTOR inhibitors. An in vitro study showed that the expression of fibronectin, COL1A1, and αSMA was upregulated by TGF-β2 treatment of hTM cells; such upregulation was significantly suppressed by mTOR inhibitors. The inhibitors significantly reduced the migration rate of TGF-β2-stimulated hTM cells. mTOR inhibitors may usefully reduce the fibrotic response of hTM cells and we may have to explore if it is also effective in in vivo model.

## Introduction

Glaucoma is known to be the second leading cause of blindness worldwide. Aberrant increases in intraocular pressure (IOP) characterize glaucoma, and such an increase in IOP can damage the optic nerve^1–3^. IOP reduction is the only established effective therapy that suppresses visual impairment and blindness in both hypertensive and normotensive individuals. IOP elevation is reported to be principally the result of increased aqueous humor (AH) outflow resistance within the conventional outflow pathway^1,4,5^. Damage to the collector channels is implicated as a cause of resistance within the distal pathway in patients with advanced glaucoma^6^. In the conventional pathway, cellular responses is reported to be involved in the pathophysiology of glaucoma. Remodeling and excess accumulation of extracellular matrix (ECM) materials, adhesive interactions, regulation of the contractile properties of trabecular meshwork cells, and decreased permeability in stratum corneum have been associated with glaucomatous degeneration of outflow pathway tissues^7–9^.

Various aqueous soluble mediators (i.e. vascular endothelial growth factor (VEGF), connective tissue growth factor (CTGF), transforming growth factor-beta (TGF-β), monocyte chemotactic protein-1 (MCP-1), and members of the matrix metalloproteinase (MMP) family), are reported to have important roles in the ECM production and induction of fibrogenic changes in human trabecular meshwork (hTM) cells. The materials act in both autocrine and paracrine manners^7,10–12^.

Mammalian target of rapamycin (mTOR) is a downstream factor affected by TGF-β, and it triggers many bioactive changes, including fibrosis^13^. Anti-fibrotic effects of mTOR inhibitors have been extensively reported in patients with hepatic or pulmonary fibrosis and rheumatic diseases^14–17^; however, to the best of our knowledge, no report has yet discussed the potential effects of mTOR inhibitors on hTM cells. The PI3K/mTOR pathway has been implicated in initiating fibroblast proliferation, survival and differentiation. The downstream cascades of PI3K is known to be regulated by the lipid phosphatase and tensin homolog (PTEN). In fibroblasts derived from lung or hepatic fibrosis patients’ tissue, they are known to show aberrant PI3K activity^18–21^. And the issue if this cascade is upregulated in glaucomatous hTM needs further exploration. In normal human tissue, the β1 integrin binds polymerised collagen and activates PTEN activity, leading to the inhibition of their proliferation. As such, targeting mTOR in those diseases could be the novel treatment in such disease, and for the glaucoma patients, we speculated that inhibition of mTOR could be a novel treatment in glaucoma patients. TGF-β-mTOR pathway is implicated in fibrotic diseases. PI3K-mTOR pathway could be involved in fibrotic changes in glaucomatous lamina cribrosa cells. Given the role of TM's profibrotic changes in contributing to elevated intraocular pressure, this study investigated whether TGF-β2-PI3K-mTOR pathway is involved in hTM cell profibrosis'.

Here, we evaluated if mTOR inhibitors might attenuate hTM cells’ fibrosis induced by TGF-β2 in vitro.

## Results

### Characterization of hTM cells

Primary hTM cells were isolated from human donor eyes, characterized as described previously by Keller et al.^22^ Supplemental Fig. 1 shows the result of immunocytochemistry to characterize the hTM cells, and the cells were positive for AQP (Aquaporin)-1, Vimentin, TIMP (Tissue Inhibitor of Metalloproteinase)-3, COL4A1 (Collagen IV Alpha 1 Chain) and MGP (Matrix Gla Protein) but were negative for desmin. And in Supplementary Fig. 2, hTM cells treated with 100 nM and 500 nM Dexamethasone showed significant upregulation of myocilin mRNA with RT-qPCR (Supplemental Fig. 2A; P < 0.01 between control and 100 nM or 500 nM Dexamethasone treated groups, and P < 0.05 between 100 and 500 nM Dexamethasone treated groups), and also significant upregulation of myocilin was confirmed with WB when hTM cells were treated with 100 nM Dexamethasone (P < 0.01, Supplemental Fig. 2B,C).

**Figure 1:**
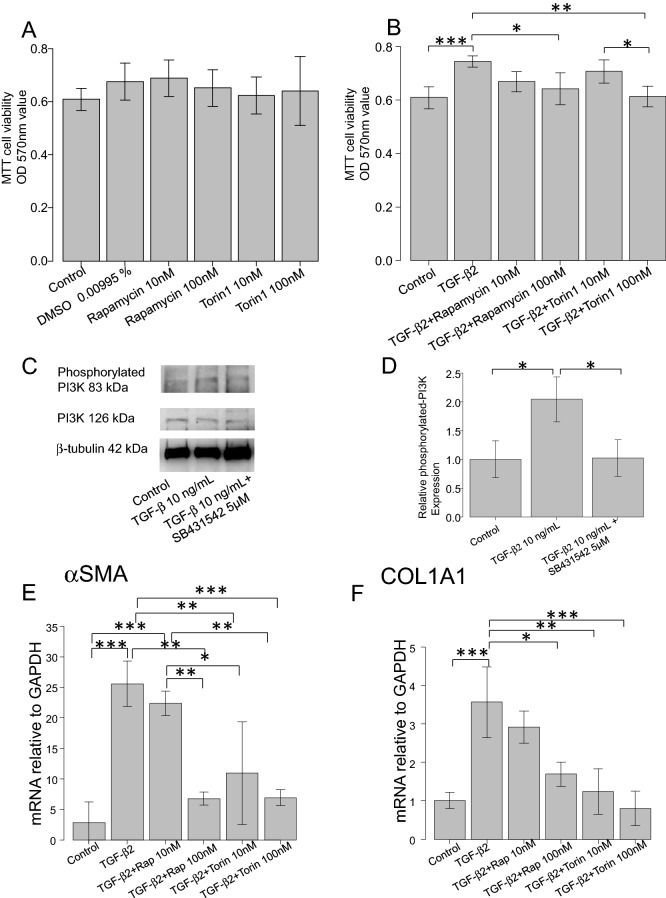
(**A**) and (**B**) Evaluation of the potential toxicities of compounds used in the hTM cells’ experiments and the effects of TGF-β2 and mTOR inhibitors on cell proliferation. (**A**) hTM cells’ toxicities after 24 h (n = 4). No compound was toxic. (**B**) Cell proliferation of hTM cells to 24 h (n = 4). Proliferation was significantly accelerated by TGF-β2 treatment (P < 0.001) and was suppressed by mTOR inhibitors (P < 0.05, P < 0.01). One-way analysis of variance followed by the Tukey *post-hoc* test. *P < 0.05, **P < 0.01, and ***P < 0.001. (**C**) and (**D**) Upregulation of the hTM cells’ PI3K-mTOR pathway by TGF-β2. (**C**) The expression level of phosphorylated-PI3K on treatment with 10 ng/mL of TGF-β2 with or without TGF-β inhibitor (5 μM SB431542) (n = 3). The results are expressed relative to that of the loading control (β-tubulin). TGF-β2 upregulated phosphorylated-PI3K in hTM cells, and the change was attenuated with TGF-β inhibitor (5 μM SB431542). One-way analysis of variance followed by the Tukey *post-hoc* test. *P < 0.05. (**E**) and (**F**) qPCR quantification of the mRNAs encoding αSMA and COL1A1 in hTM cells relative to the GAPDH level (n = 4). The relative mRNA expression levels of (**A**) αSMA and (**B**) COL1A1 were significantly higher (compared to the control values) on stimulation with TGF-β2, and the increases were reduced by the mTOR inhibitors rapamycin and Torin 1. One-way analysis of variance followed by the Tukey *post-hoc* test. *P < 0.05, **P < 0.01, ***P < 0.001.

**Figure 2:**
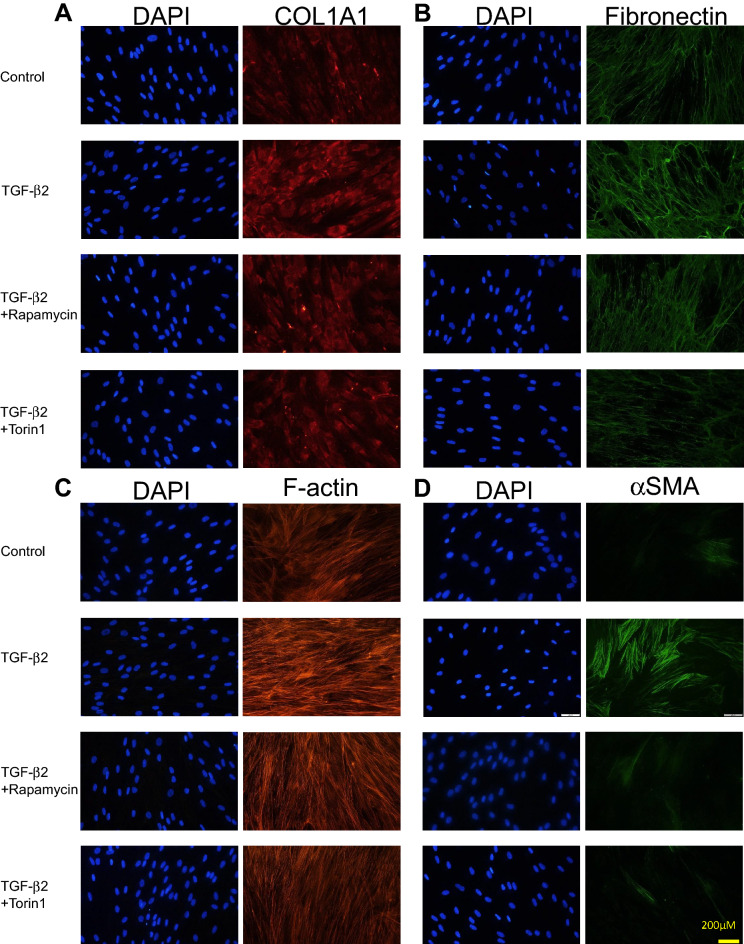
Immunocytochemistry detecting COL1A1, fibronectin, F-actin, and αSMA in hTM cells. The first panel shows cells that were not treated with TGF-β2 or mTOR inhibitors. The second panel shows cells treated with TGF-β2. The third panel show cells treated with TGF-β2 and 100 nM rapamycin. The fourth panel shows cells treated with TGF-β2 and 100 nM Torin 1. Cells stained with DAPI are shown by the left rows, and the staining levels of COL1A1 (**A**), fibronectin (**B**), F-actin (**C**), and aSMA (**D**) are shown by the right rows. These materials were upregulated in hTM cells exposed to TGF-β2, and the fibrotic responses were significantly suppressed by the mTOR inhibitors rapamycin and Torin 1.

### Effects of TGF-β2 and the mTOR inhibitors on cell proliferation

First, we explored the potential toxicity of DMSO (0.00995% v/v), rapamycin (10 nM or 100 nM), and Torin 1 (10 nM or 100 nM) toward hTM cells using the MTT assay. Figure 1A shows that there was no significant difference among the groups, and also no significant difference between the control and DMSO-treated groups. As shown in Fig. 1B, the OD_570_ value increased significantly when the cells were treated with TGF-β2 (10 ng/mL) compared to the control group (P < 0.001). The TGF-β2-induced upregulated OD_570_ value was significantly attenuated in the presence of 100 nM rapamycin (P < 0.05) or 100 nM Torin 1 (P < 0.01).

### Upregulation of PI3K-mTOR signaling induced by TGF-β2

We next assessed if PI3K-mTOR signaling was upregulated by TGF-β2. Figure 1C, D (n = 3) show the Western blots used to assess induction of the phosphorylation of PI3K cascade in response to 10 ng/mL of TGF-β2 with or without TGF-β inhibitor (SB431542 5 μM). Phosphorylated-PI3K expression was upregulated by TGF-β2, and was inhibited with 5 μM SB431542 (Fig. 1C). A significant difference was evident between the control and TGF-β2-treated groups (P < 0.05) and TGF-β2-treated group and SB431542 pre-treated groups (P < 0.05) (Fig. 1D).

### The effects of TGF-β2 and mTOR inhibitors on the expression of mRNAs encoding alpha-smooth muscle actin (αSMA) and COL1A1 (Collagen I Alpha 1 Chain) in hTM cells

The mRNA levels in hTM cells were assessed by qPCR (Fig. 1E, F). The mRNA expression levels of αSMA and COL1A1 were significantly higher after the addition of TGF-β2 than those of the control group (P < 0.001), but these rises were reduced by the mTOR inhibitors (Fig. 1E; P < 0.01 for 100 nM Rapamycin and 10 nM Torin1, and P < 0.001 for 100 nM Torin1, Fig. 1F; P < 0.05 for 100 nM Rapamycin, P < 0.01 for 10 nM Torin1, P < 0.001 for 100 nM Torin1).

### The effects of TGF-β2 and mTOR inhibitors on the fibrotic responses of hTM cells as assessed via immunohistochemistry and Western blotting

**Figure 3:**
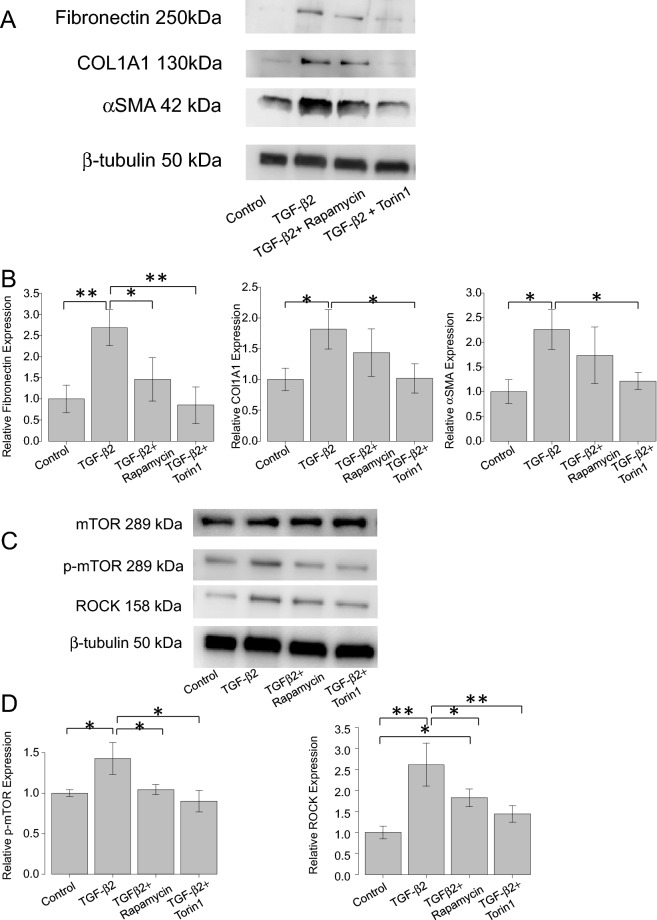
(**A**) and (**B**) Western blotting for fibronectin, COL1A1, F-actin, and αSMA in hTM cells. The representative bands for fibronectin, COL1A1, and αSMA are shown in (**A**), and the relative expression of fibronectin, COL1A1, and αSMA compared to the loading control (β-tubulin) are shown in (**B**) (n = 3). The various components were upregulated in hTM cells exposed to TGF-β2, and the fibrotic responses were significantly suppressed by the mTOR inhibitors rapamycin and Torin 1. One-way analysis of variance followed by the Tukey *post-hoc* test. *P < 0.05, **P < 0.01. (**C**) and (**D**) Western blotting for mTOR, phosphorylated mTOR and ROCK1 + ROCK2 in hTM cells. The representative bands for fibronectin, mTOR, phosphorylated-mTOR and ROCK1 + ROCK2 are shown in (**C**), and the relative expression of fibronectin, mTOR, phosphorylated-mTOR and ROCK1 + ROCK2 compared to the loading control (β-tubulin) are shown in (**D**) (n = 3). The various components were upregulated in hTM cells exposed to TGF-β2, and the fibrotic responses were significantly suppressed by the mTOR inhibitors rapamycin and Torin 1. One-way analysis of variance followed by the Tukey *post-hoc* test. *P < 0.05, **P < 0.01.

Immunocytochemistry and Western blotting were used to assess the fibrotic responses to TGF-β2 and the effects of the mTOR inhibitors (rapamycin and Torin 1). Figure 2 shows the immunocytochemical data. The levels of COL1A1, fibronectin, phalloidin, and αSMA increased in hTM cells after TGF-β2 treatment, and these fibrotic responses were significantly suppressed by rapamycin or Torin 1. Figure 3A, B shows the Western blotting data (n = 3), which also indicate that the levels of fibronectin, COL1A1, and αSMA were upregulated in hTM cells after TGF-β2 treatment (P < 0.01 for Fibronectin, P < 0.05 for COL1A1 and αSMA), and that these fibrotic responses were significantly suppressed by Torin1 (P < 0.01 for Fibronectin, P < 0.05 for COL1A1 and αSMA). Also, the expression of fibronectin upregulated with TGF-β2 treatment was significantly attenuated with rapamycin (Fig. 3A, B; P < 0.05).

### Upregulation of mTOR and ROCK (Rho-Associated Kinase) 1 + ROCK2 induced by TGF-β2 and its attenuation with rapamycin or Torin1

Figure 3C, D shows the expression of mTOR, phosphorylated-mTOR, ROCK1 + ROCK2 when hTM cells were stimulated under TGF-β2 with or without rapamycin or Torin1. As shown in Fig. 3C, D, the expression of phosphotylated-mTOR and ROCK1 + ROCK2 was significantly upregulated with TGF-β2 stimulation (Fig. 3C, D; P < 0.05 for phosphorylated-mTOR and P < 0.01 for ROCK1 + ROCK2), and the change was attenuated with rapamycin or Torin1 (Fig. 3D; P < 0.05 for both Rapamycin and Torin1 for phosphorylated-mTOR and, P < 0.05 for Rapamycin and P < 0.01 for Torin1 for ROCK).

### Effects of TGF-β2 and mTOR inhibitors on cell migration

**Figure 4:**
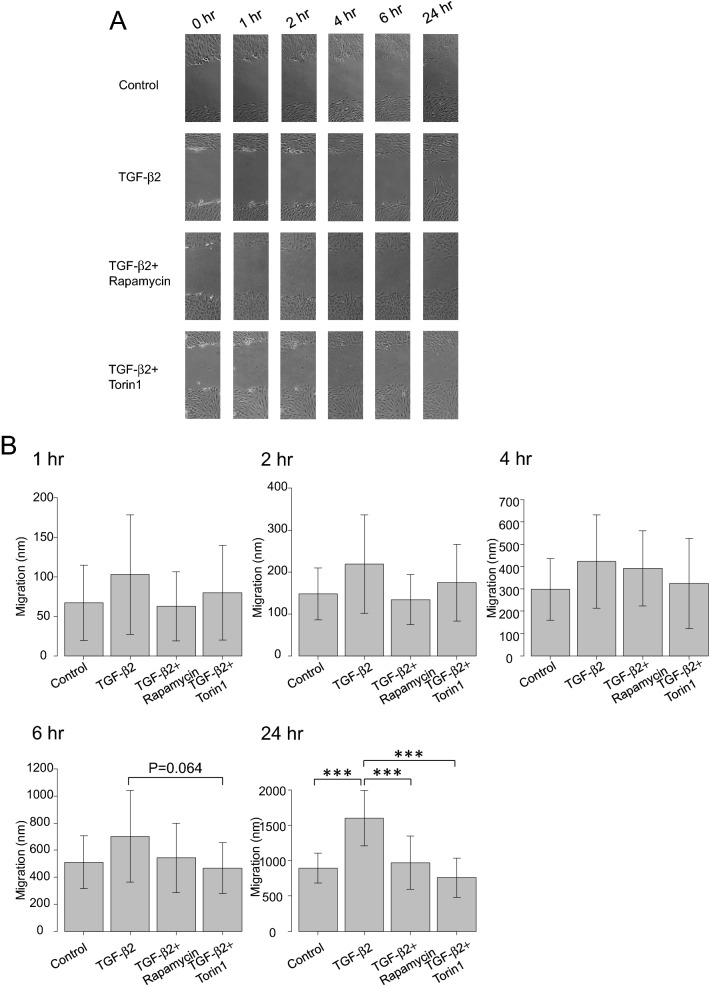
Effect of TGF-β2 and mTOR inhibitors on cell migration. (**A**) Representative phase-contrast images of scratched hTM cells. The shortest distances between the edges of migrated cells (including their protrusions) were measured from both sides. (**B**) Migration of hTMs over 1, 2, 4, 6, and 24 h (n = 15). The baseline indicates the migration distance of cells not exposed to TGF-β2 or the mTOR inhibitors. TGF-β2 significantly accelerated migration to 24 h (P < 0.001); this was suppressed by mTOR inhibitors (P < 0.001). One-way analysis of variance followed by the Tukey *post-hoc* test. ***P < 0.001.

We next performed a cell migration assay to examine the effect of TGF-β2 on hTM cells’ motility (Fig. 4). hTM cells’ migration after 1, 2, 4, 6, and 24 h was recorded; TGF-β2 significantly accelerated migration to 24 h (P < 0.001) and mTOR inhibitors suppressed this TGF-β2-mediated effect (P < 0.001).

## Discussion

We explored whether mTOR inhibitors reduced ECM remodeling and TM cell fibrosis, both of which are thought to play roles in the pathogenesis of glaucoma. Many soluble mediators affect ECM remodeling and TM fibrosis; in particular, elevated levels of TGF-β2 play a critical role in glaucoma pathogenesis. Although the fibrogenic changes induced by TGF-β2 are thought to be crucial in terms of IOP elevation and downregulation of AH drainage, the detailed mechanism is not fully understood. Many efforts have been made to reduce fibrogenic changes, and ROCK inhibitors are currently reported as a new treatment to reduce ECM remodeling or TM fibrosis. And alongside with the ROCK inhibitors, novel treatments or medications are urgently needed for those who are refractive to the existing treatments. We focused on the PI3K-mTOR pathway, which is involved in many biological processes, including cell proliferation and fibrosis, to explore if inhibition of the cascade of PI3K-mTOR pathway could aid glaucoma treatment.

In our in vitro study, TGF-β2 stimulation upregulated the expression of phosphorylated-PI3K (Fig. 1C, D, P < 0.05), and the change was attenuated with TGF-β inhibitor (Fig. 1C, D, P < 0.05), supporting the involvement of the PI3K-mTOR pathway in glaucoma pathogenesis. PI3K-mTOR pathway dysfunction has been implicated in many fibrotic diseases, including cancer and pulmonary or cardiac fibrosis. Overexpression of components of the PI3K-mTOR pathway often dysregulate cell proliferation, apoptosis, inflammation, and autophagy, triggering fibrosis progression^23,24^. Recently, Irnated et al.^25^ reported elevated levels of mRNA encoding PI3K-mTOR in glaucomatous lamina cribrosa cells. To the best of our knowledge, ours is the first study to report the involvement of the PI3K-mTOR pathway in the glaucomatous changes of hTM cells.

To explore if inhibition of the cascade of PI3K-mTOR pathway reduced the fibrogenic changes in hTM cells, we conducted additional in vitro studies to determine the effects of mTOR inhibitors on the fibrotic changes in hTM cells (Figs. 1E, F, 2, 3A, B). TGF-β2 significantly induced the expression of αSMA, COL1A1, and fibronectin by hTM cells, as revealed by qPCR (Fig. 1E, F, P < 0.001) and immunocytochemistry (Fig. 2) and Western blotting (Fig. 3A, B; P < 0.01 for Fibronectin, P < 0.05 for COL1A1 and αSMA). The mTOR inhibitors (rapamycin and Torin 1) significantly suppressed these fibrotic responses in qPCR (Fig. 1E; P < 0.01 for 100 nM Rapamycin and 10 nM Torin1, and P < 0.001 for 100 nM Torin1, Fig. 1F; P < 0.05 for 100 nM Rapamycin, P < 0.01 for 10 nM Torin1, P < 0.001 for 100 nM Torin1), in immunocytochemistry (Fig. 2), and western blotting (Fig. 3A, B; Torin1 showed significance of P < 0.01 for Fibronectin, P < 0.05 for COL1A1 and αSMA and rapamycin showed significance in inhibiting the expression of fibronectin by P < 0.05), suggesting that inhibition of mTOR reduces hTM fibrosis, ECM remodeling, and the associated fibrogenic changes. There exists two enzyme complexes; mTOR complex 1 and 2 (mTORC1 and mTORC2), and Rapamycin is a mTORC1 inhibitor and mTORC2 is known to be insensitive to rapamycin. Torin1 is an ATP-competitive mTOR inhibitor which directly inhibits both mTORC1 and mTORC^15^. As such, we speculated that rapamycin didn’t show enough effect on attenuation of TGF-β2 induced fibrogenic changes compared to Torin1.

In our in vitro study, TGF-β2 stimulation upregulated the expression of phosphorylated-mTOR and ROCK1 + ROCK2 in parallel with the expression of αSMA (Fig. 3C, D; P < 0.05 for phosphorylated-mTOR and P < 0.01 for ROCK1 + ROCK2), and the change was also attenuated with rapamycin or Torin1 (Fig. 3D; P < 0.05 for both Rapamycin and Torin1 for phosphorylated-mTOR and, P < 0.05 for Rapamycin and P < 0.01 for Torin1 for ROCK).

Also, as shown in Fig. 4, TGF-β2 significantly accelerated cell migration to 24 h (P < 0.001); such migration was significantly attenuated by the mTOR inhibitors (P < 0.001). The mechanisms regulating AH outflow and IOP are heavily dependent on physiological changes in the cytoskeleton and ECM surrounding hTM cells. In glaucoma patients, impaired cell shape, actin cytoskeleton, and cell adhesive interactions, are observed in TM cells^7^. And in in vivo rat model, such impairment observed in TM cells it induced an increase in TM contractile activity, a decrease in AH outflow facility, and elevated the IOP and fibrogenic activity of TM^7^. As such, aberrant migration or proliferation of the hTM cells may contribute to hTM cell dysfunction and cause upregulation of IOP. Referring to those reports in the past^7,11,12^, inhibition of cell migration or proliferation could benefit in glaucoma treatment.

Our work has several limitations. First, we produced only in vitro data; in vivo studies to assess the effectiveness of mTOR inhibitors in a glaucoma model are required. Second, mTOR inhibitors should be tested in clinical trials. Third, this is an in vitro study to modify the pathogenesis of POAG by stimulating normal hTM cells with TGF-β2, further in vitro studies using glaucomatous hTM cells without using any glaucomatous stimuli like TGF-β2.

When treating glaucoma patients, the attainment of a sustained reduced IOP is the only known method, but some patients are refractive to existing glaucoma eyedrops. In addition to further exploration of the pathogenesis of glaucoma, new glaucoma eyedrops which can modulate those proven mechanisms are required. It has been suggested that glaucomatous fibrotic hTM changes are associated with a number of factors that drive fibrosis, including TGF-β2, as explored in the present study. The various factors may affect hTM cells via a multitude of downstream signaling pathways, including the PKCα, MAPK, Rho-Rho kinase, JNK, and (possibly) PI-3K/Akt-mTOR pathways. An understanding of the roles played by these pathways, and their crosstalk, might slow or prevent excessive pro-fibrotic ECM gene induction and the resulting fibrosis that is characteristic of glaucoma. We found that mTOR targeting downregulated the fibrogenic cascade; modulation of mTOR activity might inhibit excessive fibrogenic changes in hTM cells and could be a novel treatment in reducing elevated IOP.

## Materials and methods

### Cell culture and passage

The hTM cells from three donor eyes (46 years old, 52 years old, and 55 years old, without glaucoma) were used in this study, and hTM cells were cultured in growth medium containing 10% fetal bovine serum (FBS) and Antibiotic Antimycotic Solution (100 ×) (Sigma-Aldrich, St. Louis, MO, USA) at 37 °C in 5% CO_2_. Cells were seeded at 5000 cells/cm^2^ and grown to confluence. Cells from passages 3–6 were used in the experiments. The cells were treated with TGF-β2 for 24 h with or without pretreatment for 30 min with rapamycin (Fujifilm Wako Pure Chemical Corp., Osaka, Japan) or Torin 1 (Selleck Chemicals, Houston, TX, USA) as an mTOR inhibitor. This study is not deemed a Human Subject Research because cells were acquired post-mortem from de-identified donor tissues and is thus considered exempt by University of Tokyo’s Institutional Review Board (IRB). Nevertheless, all experiments were done in accordance with the tenets of the Declaration of Helsinki. And this study does not include in vivo data, but all the in vitro studies were performed in accordance with ARRIVE guideline.

### MTT assay

We used an MTT Cell Proliferation Assay Kit (BioAssay Systems, Hayward, CA, USA) to evaluate cell proliferation. The assay was performed according to the manufacturer’s protocol. Briefly, cells were plated in 96-well plates and 80 μL of culture medium with or without TGF-β2 (10 ng/mL), rapamycin (10 nM or 100 nM), Torin 1 (10 nM or 100 nM), or 0.00995% (v/v) DMSO added, followed by incubation in a CO_2_ incubator at 37 °C overnight. Then, 15 μL of the MTT reagent was added to each well, followed by gentle mixing and incubation for 4 h in a CO_2_ incubator at 37 °C. Solubilizer (100 μL) was added to each well, followed by gentle mixing on an orbital shaker for 1 h at room temperature. The absorbance at 570 nm was then recorded.

### Quantitative polymerase chain reaction (qPCR)

Cells were lysed with Isogen (Nippon Gene, Tokyo, Japan) and mRNA was isolated in chloroform/isopropyl alcohol. A PrimeScript RT Reagent Kit (Takara Bio, Shiga, Japan) was used to synthesize cDNA. qPCR was performed as described previously^26^. The primer sequences for GAPDH and fibronectin have been published; all primers were purchased from Hokkaido System Science (Hokkaido, Japan).

The sequences of the PCR primers were: GAPDH, forward 5′-GAGTCAACGGATTTGGTCGT-3′ and reverse 5′-TTGATTTTGGAGGGATCTCG-3′; αsma forward 5′-CCGACCGAATGCAGAAGGA-3′ and reverse 5′-ACAGAGTATTTGCGCTCCGAA-3′; and collagen type I, alpha 1 chain (COL1A1) forward 5′-CAGCCGCTTCACCTACAGC-3′ and reverse 5′-TTTTGTATTCAATCACTGTCTTGCC-3′; Myocilin, forward 5′-TACACGGACATTGACTTGGC-3′ and reverse 5′-ATTGGCGACTGACTGCTTAC-3′. The data were normalized relative to the GAPDH amplification level.

### Immunocytochemistry

Cells were grown in chamber slides. After serum starvation for 24 h, the cells were treated with 10 ng/mL of TGF-β2 for 24 h with or without an mTOR inhibitor (100 nM rapamycin or 100 nM Torin 1), which was added 30 min before TGF-β2 treatment. The cells were fixed in ice-cold 4% (v/v) paraformaldehyde for 15 min, permeabilized with 0.3% (v/v) Triton X-100 for 5 min, and blocked in 3% (w/v) bovine serum albumin for 30 min. Immunocytochemistry was performed as described previously^26^. The primary antibodies were anti-fibronectin (1:400; Abcam, Cambridge, MA, USA), anti-collagen type I (1:400; Cell Signaling Technology, Danvers, MA, USA), anti-rhodamine phalloidin (7:1000; Thermo Fisher Scientific, Waltham, MA, USA), and anti-αSMA (Sigma-Aldrich), anti-AQP-1 antibody (1:500; Santa Cruz Biotechnology, Inc., Santa Cruz, CA), anti-vimentin antibody (1:1000; Abcam, Cambridge, MA), anti-desmin antibody (1:200; Abcam), anti-TIMP-3 antibody (KYOWA PHARMA CHEMICAL, CO., LTD, Toyama, Japan), anti-COL4A1 antibody (OriGene Technologies Inc., Rockville, MD, USA), and anti-MGP antibody (1:500; Santa Cruz Biotechnology, Inc., Santa Cruz, CA). Alexa Fluor 488- and 594-tagged secondary antibodies (1:1000) were purchased from Thermo Fisher Scientific.

### Western blotting

Cells were first starved by incubation for 24 h in serum-free medium and then treated with 10 ng/mL of TGF-β2 for 24 h with or without an mTOR inhibitor (100 nM rapamycin or 100 nM Torin 1) or TGF-β inhibitor (SB431542 5 μM), which was added 30 min before TGF-β2 treatment commenced. The cells were then collected in RIPA Buffer (Thermo Fisher Scientific K.K., Kanagawa, Japan) containing protease inhibitors (Roche Diagnostics, Basel, Switzerland), sonicated, and centrifuged. The protein concentrations in the supernatants were determined by a BCA assay using a BCA Protein Assay Kit (Thermo Fisher Scientific K.K.). Western blotting was performed as described previously^26^. 20 μg of protein per well was loaded on the gel. Protein bands were detected using the ImageQuant LAS 4000 Mini-System (GE Healthcare, Chicago, IL, USA). The primary antibodies were anti-αSMA (Sigma-Aldrich; 1:1000), anti-fibronectin (1:1000; Abcam), anti-collagen type I (1:1000; Cell Signaling Technology), Anti-Phospho-PI3-Kinase p85 antibody (1:1000, Merck), Anti-PI3K antibody (1:1000, Abcam), Anti-ROCK1 + ROCK2 antibody (1:1000, abcam), mTOR antibody (1:1000, Cell Signaling Technology), anti-myocilin (NT) antibody (1:1000,  Merck Millipore), phospho-mTOR antibody (1:1000: Cell Signaling Technology). The horseradish peroxidase (HRP)-conjugated second antibody was purchased from Thermo Fisher Scientific. β-Tubulin served as the loading control. All membranes were stripped of antibodies using Western blotting stripping solution and incubated with mouse monoclonal antibodies against β-tubulin (1:1000, Wako Pure Chemical Industries, Ltd) and then with HRP-conjugated goat anti-mouse or rabbit IgG antibodies (1:2000). Densitometric film analysis was performed with the aid of ImageJ ver. 1.49 (NIH, Bethesda, MD, USA) and the results expressed relative to the intensity of the loading control (β-tubulin).

### Migration assay

Cells were starved by incubation for 24 h in serum-free medium. Four scratches were made in each well with a pipette tip and the wells were photographed under a microscope (BZ-9000; Keyence Corp., Osaka, Japan).

The starvation medium was then changed to medium containing TGF-β2 or TGF-β2 with an mTOR inhibitor at the indicated concentration. The cells were incubated at 37 °C and the scratches photographed 1, 2, 4, 6, and 24 h later. The scratch widths were the shortest distances between the edges of migrated cells (including protrusions) on both sides. These regions were measured using ImageJ ver. 1.49 software (NIH). Changes in scratch widths were recorded and used to calculate migration distances.

### Statistical analysis

Data were statistically analyzed using the EZR program (Saitama Medical Center, Hidaka, Japan)^27^. The results are expressed as means ± standard deviations (SDs). The *t*-test or Mann–Whitney U test was used to compare two variables, and the Steel–Dwass test employed to compare multiple variables. Differences among groups were analyzed by a one-way analysis of variance followed by the Tukey *post-hoc* test. P-value < 0.05 was considered statistically significant.

## Supplementary Information

Supplementary Figures.
