# Supplementary material for: mTOR inhibitors potentially reduce TGF-β2-induced fibrogenic changes in trabecular meshwork cells
Source: Sci Rep. 2021 Jul 8;11:14111. doi: 10.1038/s41598-021-93580-3 (PMC8266857; doi:10.1038/s41598-021-93580-3)
Supplement: Supplementary file 1 — Supplementary Figures. [file 41598_2021_93580_MOESM1_ESM.pdf]

mTOR inhibitors potentially reduce TGF-β2-induced fibrogenic changes in trabecular meshwork cells

Nozomi Igarashi[1], Megumi Honjo[1**], and Makoto Aihara [1].

Supplemental Figure 1

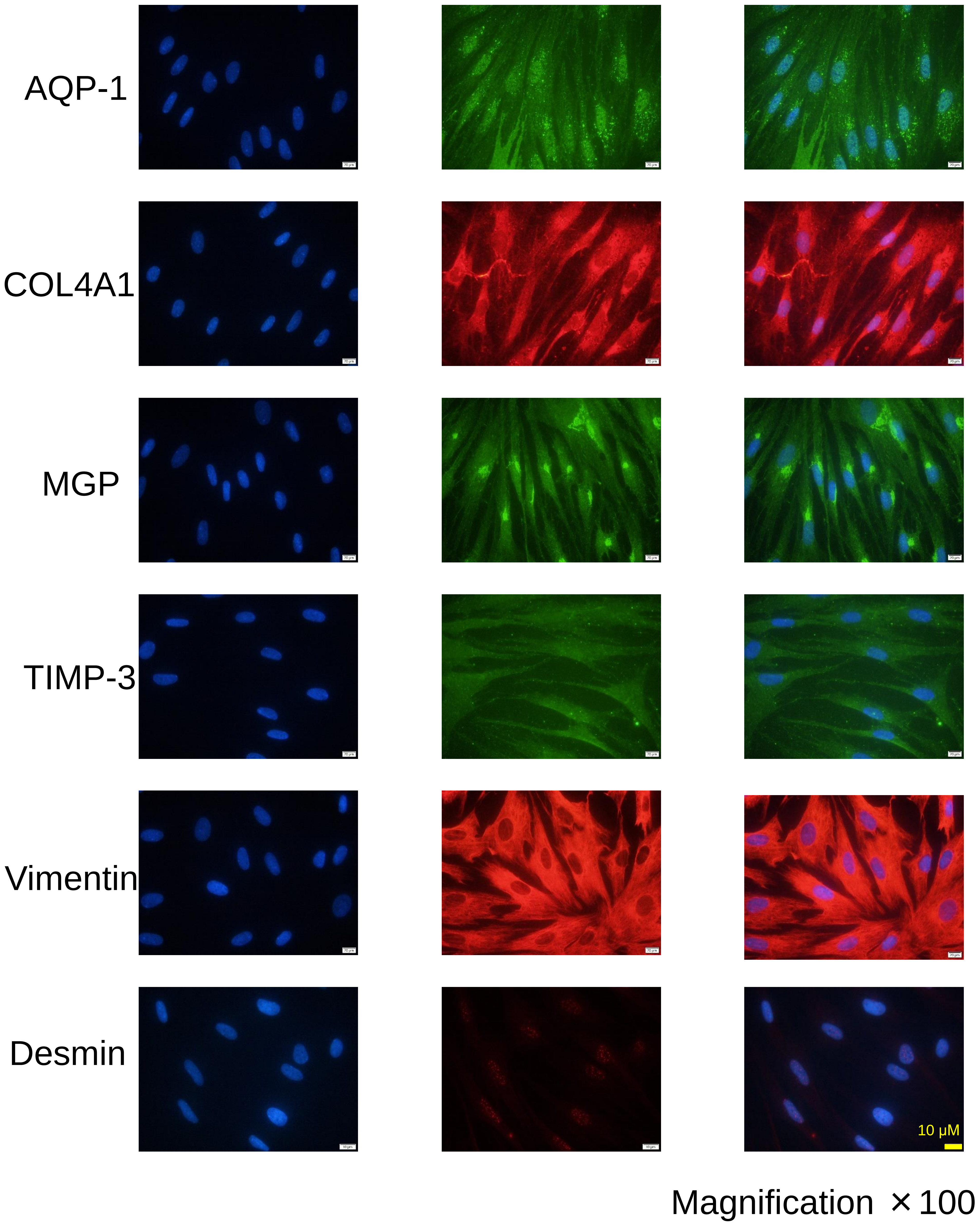

Supplemental Figure 2

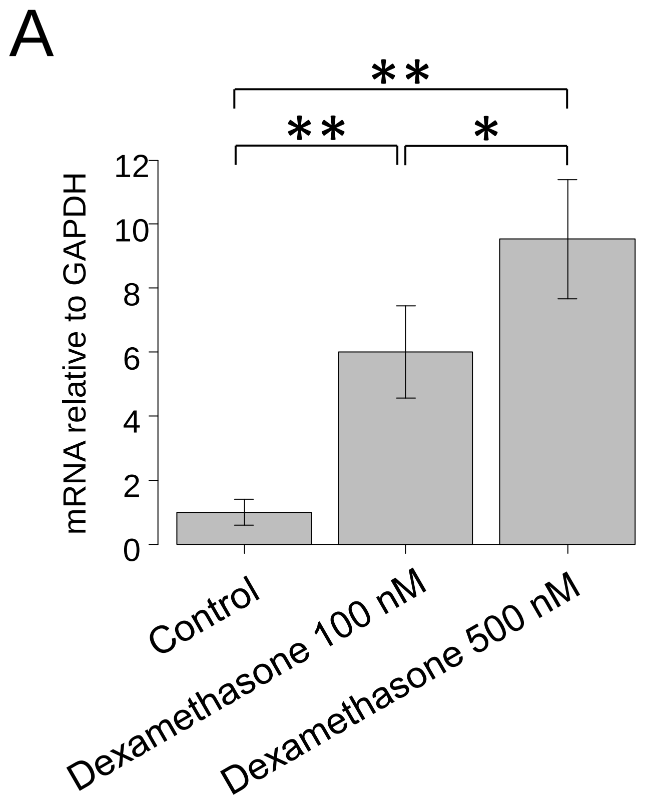

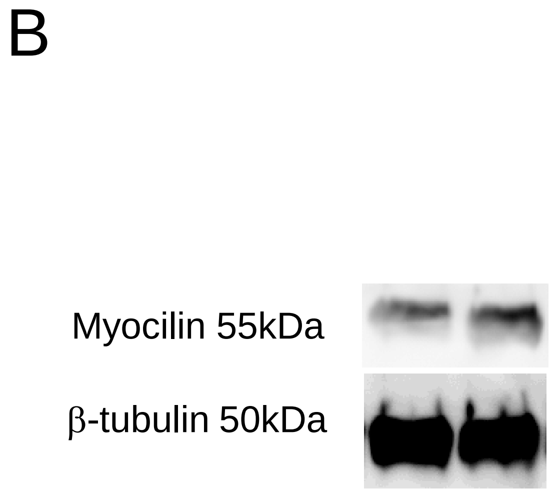

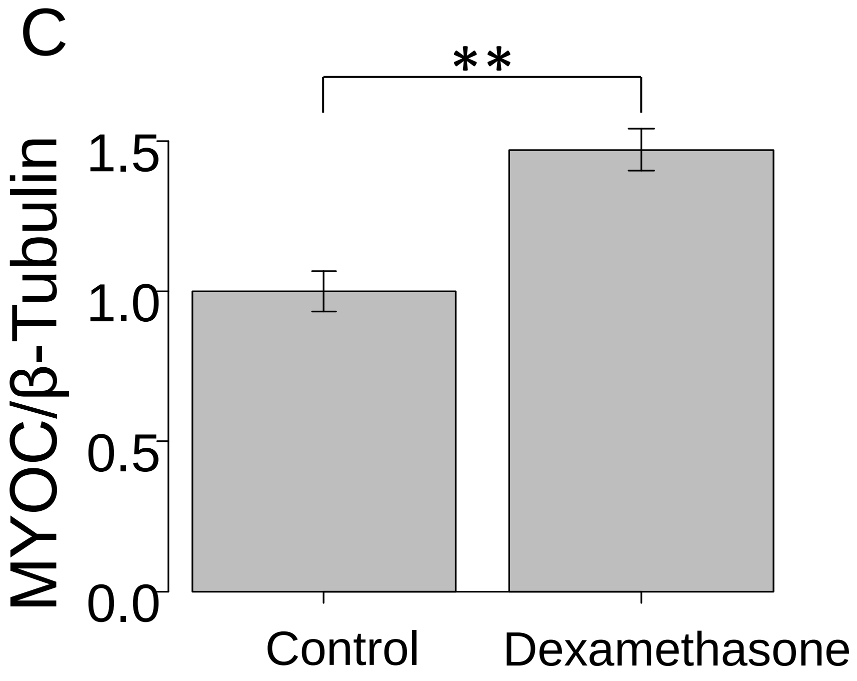

Supplementary Figure 1. Characterization of hTM cells with immunocytochemistry.

The panels on the left show cells stained for 4’,6-diamidino-2-phenylindole (DAPI; blue).

The panels on the middle shows endothelial cell markers, and mesenchymal markers, and the panels on the right shows merged images.

The hTM cells used in the study were positive for AQP-1, TIMP-3, COL4A1, MGP, vimentin, but were negative for desmin. Bar, 10 µm.

Supplemental Figure 2. Characterization of hTM cells with Western blotting and qPCR.

(A) The statistically significantly increased mRNA expression of myocilin was confirmed with 100 nM and 500 nM dexamethasone (Dex) treatment (7 days).

One-way analysis of variance followed by the Tukey *post-hoc* test. $^*p<0.05$, $^{**}p<0.01$.

(B) Upregulation of myocilin was confirmed with western blotting in hTM cells stimulated with 100 nM Dex. The representative bands for western blotting.

(C): The relative expression of myocilin to the loading control of β-tubulin ($n = 3$). T-test. $^{**}p<0.01$.

Myocilin
55kDa

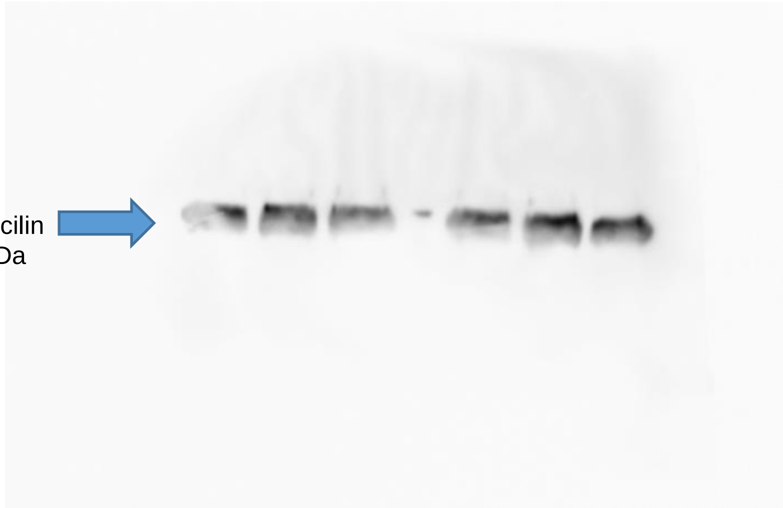

β-tubulin
50 kDa

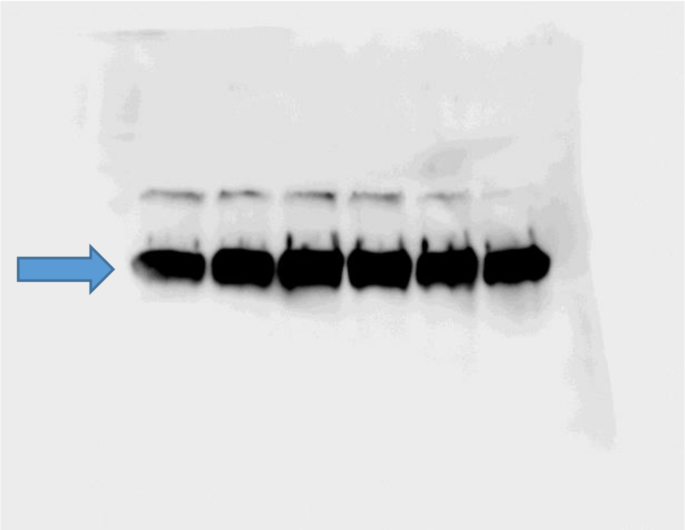

ROCK1 + ROCK 2 158 kDa

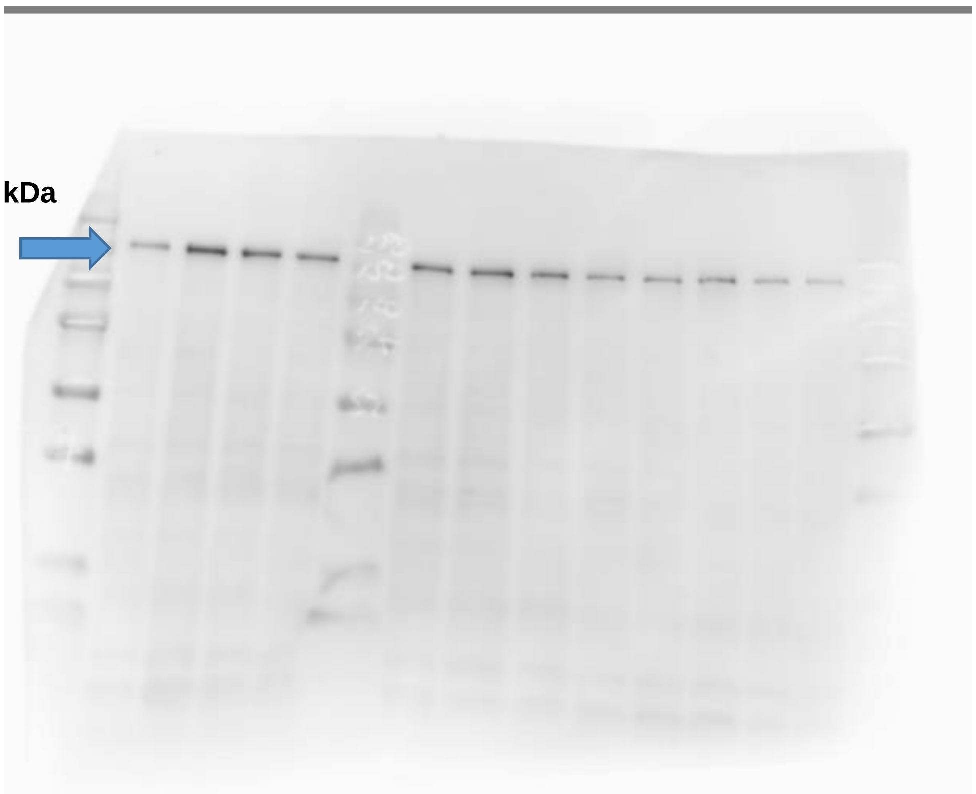

p-mTOR 289 kDa

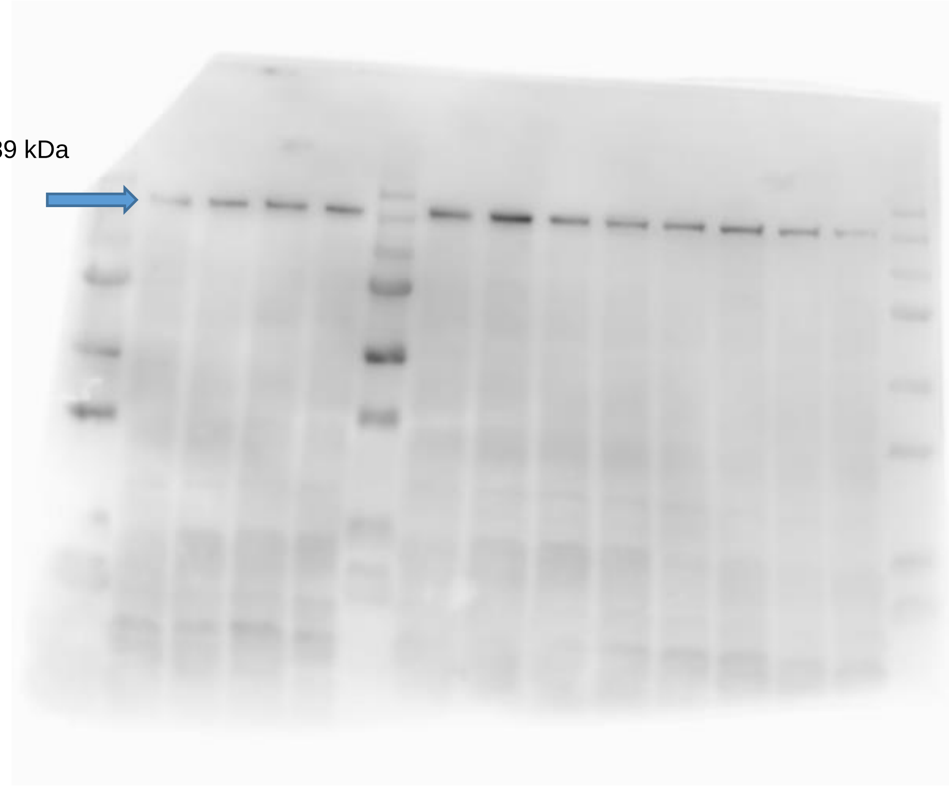

β-tubulin 50 kDa

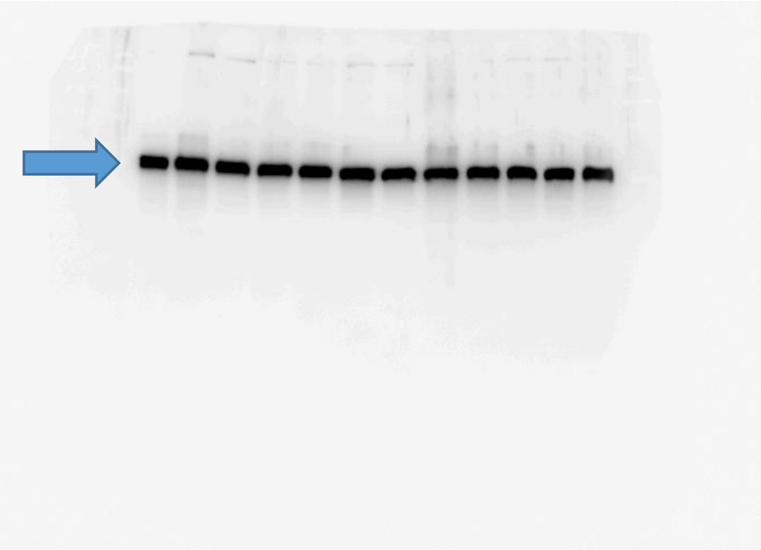

mTOR 289 kDa

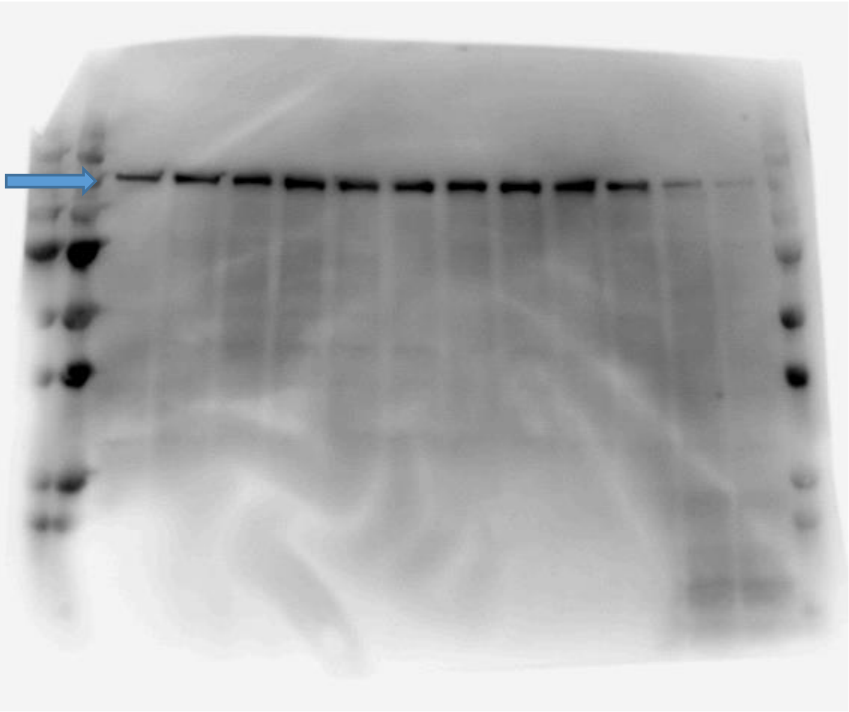

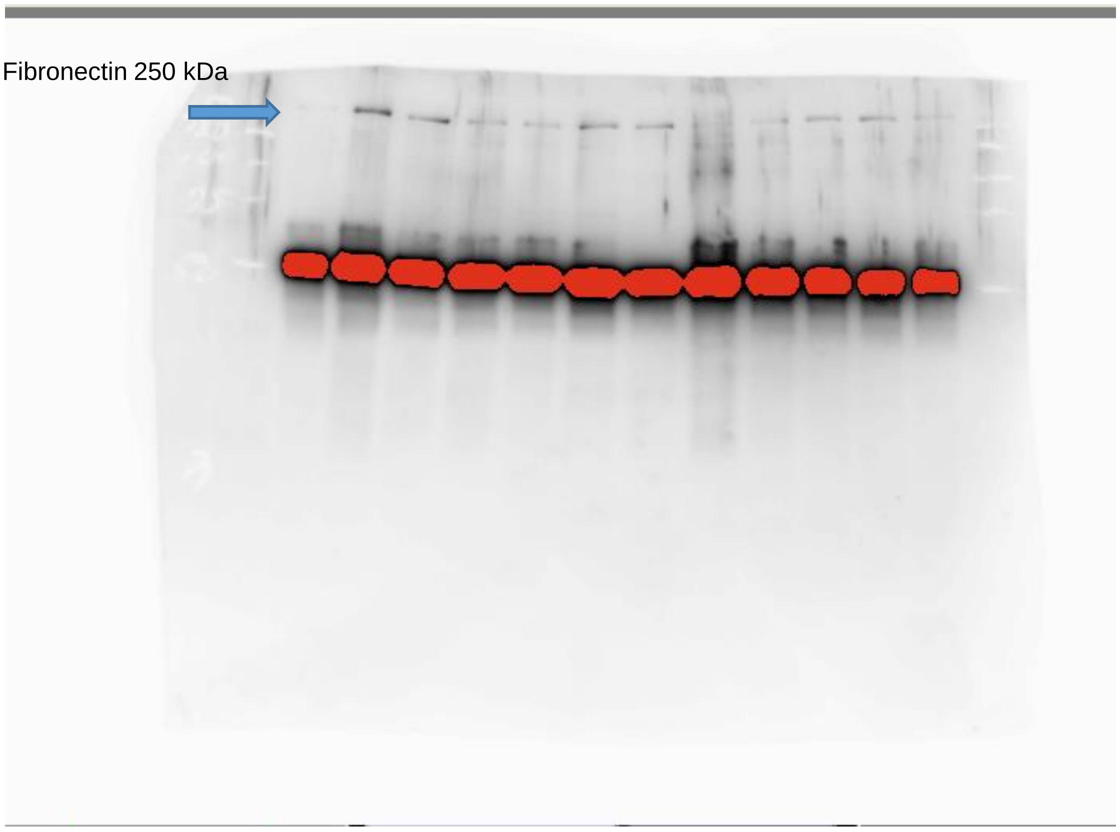

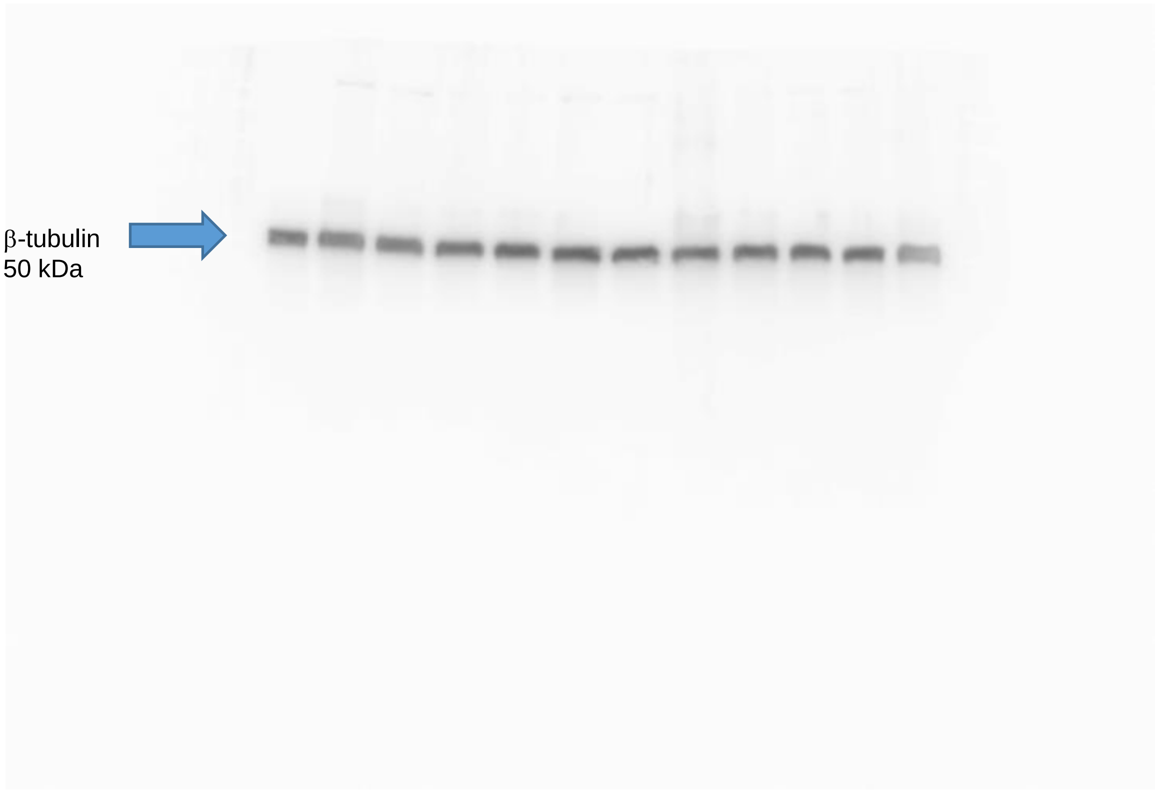

α–SMA 42 kDa

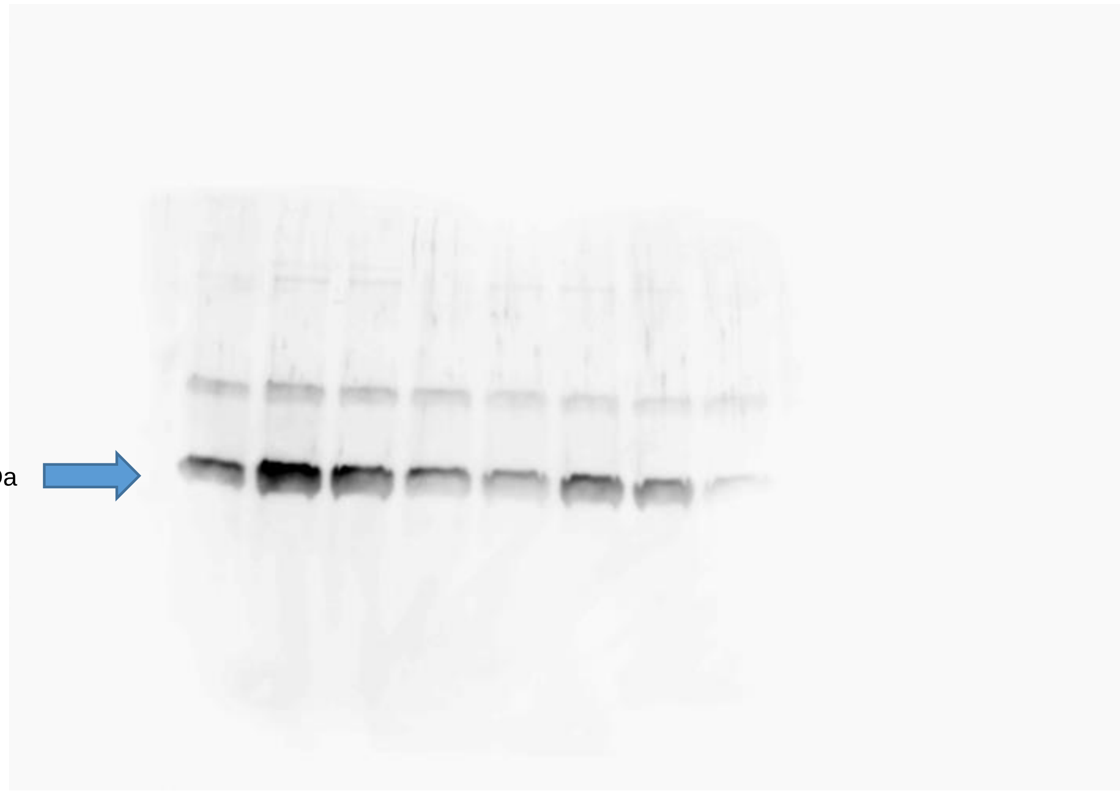

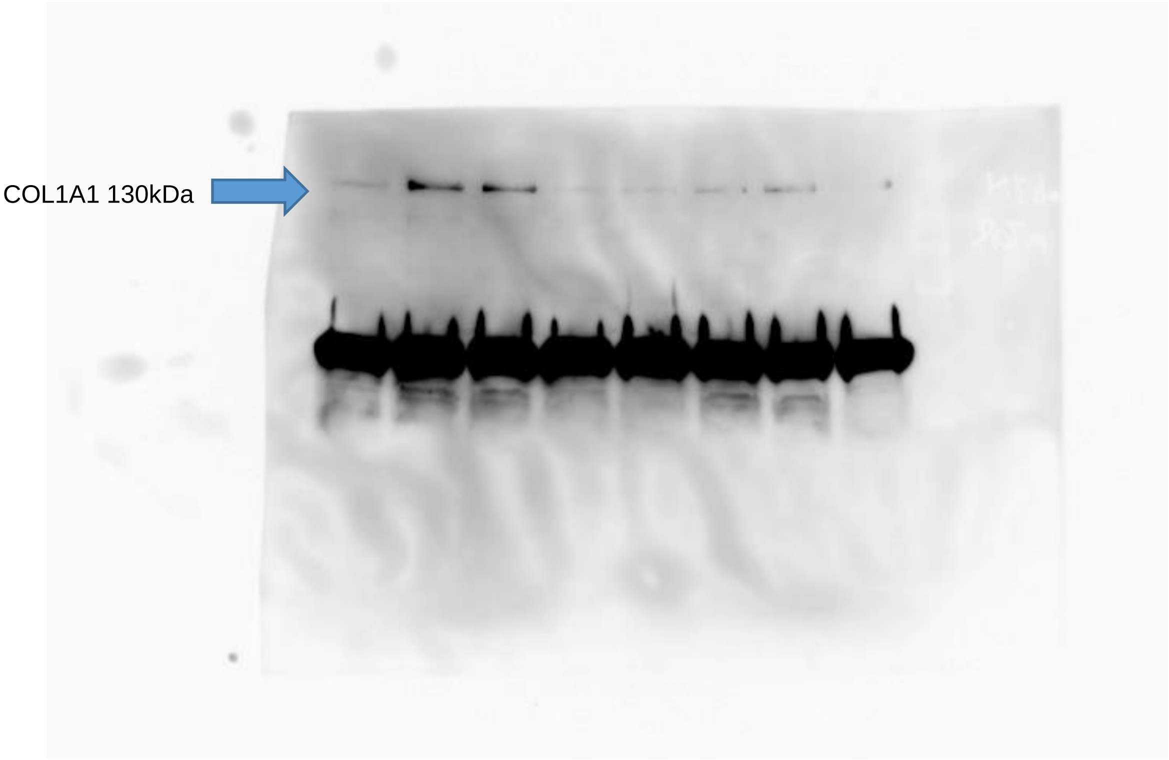
COL1A1 130kDa

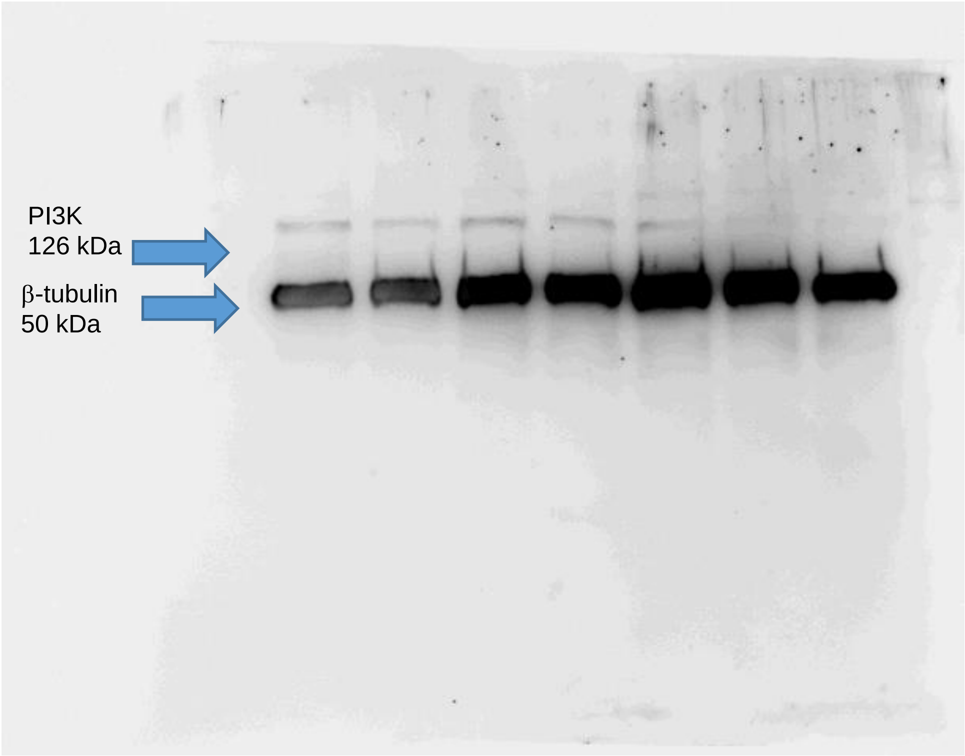
PI3K
126 kDa
β-tubulin
50 kDa

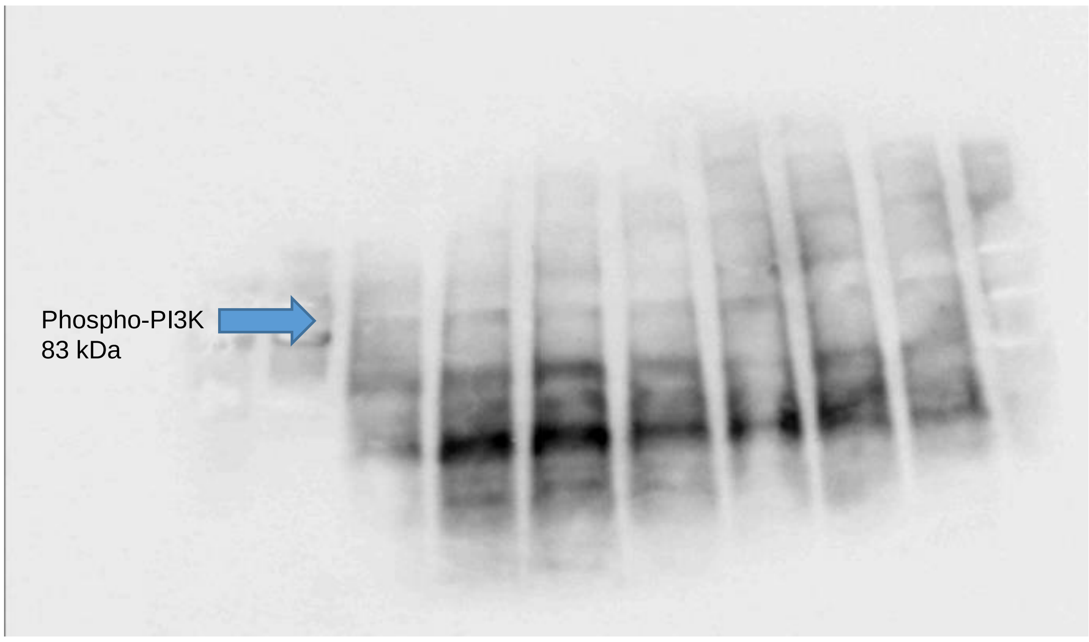
Phospho-PI3K
83 kDa
